# Neural and Genetic Bases for Human Ability Traits

**DOI:** 10.3389/fnhum.2020.609170

**Published:** 2020-12-16

**Authors:** Camila Bonin Pinto, Jannis Bielefeld, Rami Jabakhanji, Diane Reckziegel, James W. Griffith, A. Vania Apkarian

**Affiliations:** ^1^Department of Physiology, Feinberg School of Medicine, Northwestern University, Chicago, IL, United States; ^2^Center for Translational Pain Research, Feinberg School of Medicine, Northwestern University, Chicago, IL, United States; ^3^Department of Medical Social Sciences, Feinberg School of Medicine, Northwestern University, Chicago, IL, United States; ^4^Department of Physical Medicine and Rehabilitation, Feinberg School of Medicine, Northwestern University, Chicago, IL, United States; ^5^Department of Anesthesiology, Feinberg School of Medicine, Northwestern University, Chicago, IL, United States

**Keywords:** human ability, heritability, neural circuits, NIH toolbox, human connectome project (HCP)

## Abstract

The judgement of human ability is ubiquitous, from school admissions to job performance reviews. The exact make-up of ability traits, however, is often narrowly defined and lacks a comprehensive basis. We attempt to simplify the spectrum of human ability, similar to how five personality traits are widely believed to describe most personalities. Finding such a basis for human ability would be invaluable since neuropsychiatric disease diagnoses and symptom severity are commonly related to such differences in performance. Here, we identified four underlying ability traits within the National Institutes of Health Toolbox normative data (*n* = 1, 369): (1) Motor-endurance, (2) Emotional processing, (3) Executive and cognitive function, and (4) Social interaction. We used the Human Connectome Project young adult dataset (*n* = 778) to show that Motor-endurance and Executive and cognitive function were reliably associated with specific brain functional networks (*r*^2^ = 0.305 ± 0.021), and the biological nature of these ability traits was also shown by calculating their heritability (31 and 49%, respectively) from twin data.

## Introduction

Understanding differences in human ability traits, such as cognition, emotion or behavioral tendency, has been an area of great interest in the field of neuropsychology. Using such ability traits can facilitate neuropsychiatric diagnosis and treatment, as well as allow the prediction of individual risks. It can help understand contributors of different conditions, such as chronic pain (Alais et al., 2010; Vachon-Presseau et al., 2016), neurodevelopmental disorders (Barch et al., 2019), autism (Happé and Frith, 1996), stroke rehabilitation (Carlozzi et al., 2017; Johnson et al., 2017), Alzheimer's disease (Snyder et al., 2011), and other neuropsychiatric conditions (Hayden et al., 2018). While personality traits have been studied extensively (Gerlach et al., 2018), human ability, including behavioral and neurological function, is still underexplored.

Human ability, compared to personality, is less well-defined. However, the National Institutes of Health (NIH) Toolbox, which provides a unified assessment of neurologic and behavioral function, quantifies a comprehensive spectrum thereof. It combines different sets of measures, including scales for emotional, cognitive, motor, and sensory function in order to provide a full range of individual assessment, making this dataset a natural candidate for mapping out human ability. The measured response parameters have been shown to be sensitive and effective in the detection of subtle differences between participants, and the underlying ability traits described by the individual tasks, like cognitive and motor skills, are well-understood (Gershon et al., 2013). Its relevance in medical research is well-established, demonstrating utility in clinical stroke (Carlozzi et al., 2017), disability classification (Hessl et al., 2016), and brain injury recovery (Tulsky et al., 2017), for example. In addition, human ability is expected to predict real world outcomes, like socioeconomic status, education levels, and health issues, just like personality traits do (Eisenberg et al., 2019).

Beyond providing a complete basis of human ability, we investigated their biological origins. We used the Human Connectome Project HCP large-scale independent components analysis (ICA) young adult dataset (Van Essen et al., 2012) to determine whether these ability traits are represented by brain properties like morphology and functional connectivity.

Additionally, we evaluated whether heritability plays a role in determining ability traits, with the goal of estimating how much of the variation of a specific ability trait is due to environmental factors vs. genetic differences. We hypothesized that ability traits with significant correlations in brain biology would also show significant heritability, since they both point to a common, biological underpinning.

## Methods

In this section we show how we constructed the statistical models underlying the analysis and what decisions were made at each step in the process. It will become clear that each step is motivated by the data; the parameterizations are all natural to these datasets.

### Data

In our initial analysis we used the NIH Toolbox normative dataset (Gershon, 2016), which contains data from healthy subjects across their entire lifespan. Ages 17 and under are tested differently, so we focused on ages 18 and above. Additionally, we drew conclusions using data from the Human Connectome Project (HCP) 1200 young adult database (ages 18–35) (Van Essen et al., 2012). Within the HCP data, we used both the NIH Toolbox assessments and the family structured data, being able to identify twins, siblings, and half siblings. This allowed us to draw parallels to the original NIH Toolbox normative data and link it to heritability results. Besides that, also from the HCP 1200 data release, we extracted the structural and functional magnetic resonance imaging (fMRI) data.

First, the data were filtered, and only NIH Toolbox scored variables present in both datasets were included. Statistical analyses were performed age- and gender-blind (the NIH Toolbox data uses gender instead of biological sex), in addition to other demographic variables. Later on, they were used to analyze the nature of the subject clusters. The set of final variables is given in Supplementary Table 4 and is used, both in the NIH Toolbox normative dataset, as well as the HCP young adult data. Included are 31 variables within the cognition, motor, sensory, and emotion domains.

To avoid over-imputation, only participants aged 18 and above that reported more than 70% of the variables in question were included. Within the HCP dataset, all participants with quality control issues in the fMRI were excluded. 778 HCP subjects ages 18–35 remained (Supplementary Figure 1). The grip strength and dexterity measures in the toolbox are directly derived from raw scores. We use these, both in the NIH Toolbox dataset, as well as the in the HCP dataset.

After filtering, the individual scores for the datasets were scaled. The final NIH Toolbox normative data were standardized to mean zero and standard deviation of one. The HCP data were scaled by these NIH Toolbox values to provide compatibility between the two datasets: We determined the mean and standard deviation for each NIH Toolbox normative data variable and z-scored the corresponding HCP Toolbox data using these.

### Factor Analysis

Starting out, we reduced the dimensionality of the data. This reveals the underlying generalized variables and can be used to reduce noise (Revelle, 2009). We tested both principal component (PCA) and factor analysis techniques (FA) and compared them via their likelihoods. In factor analysis models, the part of the model describing the error is handled more flexibly in contrast to principal components (Hastie et al., 2009) (Supplementary Figure 2A). Principal components model the error using a covariance matrix ∝ σ^2^𝕀, where 𝕀 is the identity, while factor analysis relaxes this to $\propto diag\left(\sigma_{1}^{2},\;\sigma_{2}^{2},\ldots ,\;\sigma_{N}^{2}\right)$.

Parallel analysis (Horn, 1965) was used to obtain the optimal number of components. This approach compares the eigenvalues of covariance matrices: One for the NIH Toolbox normative data and one based on white noise. Factors corresponding to eigenvalues smaller than the ones of the noise covariance were discarded (Supplementary Figure 2B). Here, we select four factors to describe the NIH Toolbox normative dataset. This choice is on the conservative side of how many features to include. Factor analysis decompositions are unique only up to a rotation^1^ (Hastie et al., 2009), so there is some freedom in picking the exact composition of latent variables. In order to minimize overlap between those, we chose a varimax rotation (Kaiser, 1958). This maximizes the variance of the squared loadings column-wise, which leads to the most block-diagonal form of the loading's matrix. In other words, coefficients of variables that contribute to multiple latent factors are minimized. We use an off-the-shelf implementation of factor rotations in python (mvds314, 2017). These rotations have no effect on the clustering of the subjects. For the HCP data, the identical factor decomposition was performed. Supplementary Figure 2B shows that the same number of factors govern the HCP dataset.

### Clustering

Cluster analysis is used to show equivalency between the ability traits in the NIH Toolbox normative and the HCP dataset. We cluster the data using Gaussian mixture models (GMM). The benefit of using these over more immediate algorithms like k-means clustering is the generalizability thereof: Any point in factor space can uniquely and easily be mapped to a cluster. Equivalently, each point in the coordinate space is assigned a probability to lie within any cluster. Numerically, a number of Gaussian distributions is fitted to the density of data points in the four-dimensional factor space. With *K* Gaussian components its mathematical form is $p\left(\text{x}\right)=\overset{K}{\underset{k=1}{\sum }}\pi_{k}N\left(\text{x}|\mu_{k},\;\Sigma_{k}\right)$ and each Gaussian density $N\left(\text{x}|\mu_{k},\Sigma_{k}\right)$ is centered around its mean μ_*k*_ with covariance Σ_*k*_ and is weighted by π_*k*_ which allows for differently sized clusters.

We use scikit-learn to fit this model to the data (Pedregosa et al., 2011). For this approach, spherical covariance matrices are chosen to keep model complexity to a minimum.

The number of clusters needs to be determined separately. We use the Bayesian Information Criterion (BIC) (Schwarz, 1978) and the Rand index (Rand, 1971) to obtain this value. The BIC trades likelihood for model complexity and gives a quantitative result for the optimal value. The Rand index, on the other hand, is a more direct measure of clustering performance and compares different partitions of the dataset regarding whether pairs of subjects within each have been clustered together or not. An adjusted Rand index of 1 indicates perfect overlap between cluster partitions, whereas a value of 0 indicates that no pairs in one partition have been clustered together in the other partition. Details for our implementation can be found in Von Luxburg (2010).

The entire analysis was performed considering individual responses to the NIH Toolbox tasks only. We specifically excluded background information from the questionnaires like age, gender, and ethnicities. The factor analysis already generalizes the variables, and the clustering separates subjects. We compared the overall representation of these covariates along the four clusters. For age and gender, we plot the proportion of each per cluster. The same GMM model was applied to the HCP dataset. Pairwise *t*-tests were performed between the biggest outlier and the rest of the clusters. The cluster with the biggest deviation from the mean in each variable is defined as this outlier. Results are displayed in Supplementary Tables 1, 2. The cluster composition and number of clusters as degrees of freedom have been tested in different clustering methods (k-means, hierarchical) to confirm that they are independent of the actual algorithm used.

### Brain Morphology and Connectivity

The HCP provides brain morphological and connectivity data. We checked the relationships between those, and the ability traits found in the NIH Toolbox normative data. We regressed brain properties against the four latent ability traits.

As variables we included both morphological and connectivity data. The HCP FreeSurfer data (Fischl, 2012) contain about 200 variables, and the HCP provides connectivity data from ~15^2^ to ~300^2^ features—independent component (IC) correlation matrices for each participant. The squared numbers indicate that the data consists of connectivities, not ICs. Technically with *n* ICs one obtains $\frac{n\left(n-1\right)}{2}=O\left(n^{2}\right)$ unique features. Multiple levels of processing of the connectivity data are provided, of which we used the most processed “analyzed” data, in which spatial distortions have been minimized and data have been aligned across modalities and across subjects using appropriate volume-based and surface-based registration methods (Glasser et al., 2013; WU-Minn, 2017). We first obtain the best dataset used for predictive modeling from all of the following combinations: (1) Any number of independent components of the data provided (15, 25, 50, 100, 200, 300). (2) Two kinds of connectivity matrices calculated from these: netmats1 and netmats2. (3) Connectivity vs. nodal degrees, and (4) inclusion of age and gender variables. The most predictive dataset is that which yields the closest L1-regularized regression in its 30% hold-out sample. The L1-paramter is optimized in-sample (IS).

Netmats1 denotes using full normalized temporal correlation between every node timeseries and every other. This is a relatively simple approach, but has various practical and interpretational disadvantages (Smith, 2012). Netmats2 utilizes partial temporal correlations between nodes' timeseries. This aims to estimate direct connection strengths better than achieved by full correlation. To slightly improve the estimates of partial correlation coefficients, a small amount of L2 regularization is applied (setting ρ = 0.01 in the Ridge Regression netmats option in FSLNets) (Smith et al., 2014; WU-Minn, 2017). For the nodal degrees, we calculated degrees for densities of 1% up to 10% in 1% increments.

After selecting the best input data, we regress these predictors against the ability traits to obtain a predictive model. To evaluate the generalizability of our findings and the robustness of the models, the initial sample of *n* = 778 HCP participants was split into two sub-samples again: training (IS, 70%) and test (OOS, 30%), independent from the above split. For the model training and optimizing of its regularization hyperparameter, a nested cross validation was performed within the training dataset (5-fold cross validation). We modeled the data using a L1-regularized linear regression. L1 regularization helps with model parsimony through feature selection and generalizability by keeping parameters small. An optimal regularization parameter avoids over- or underfitting the model. All predictors are included simultaneously in the regression, how many of those are used depends on the size of the regularization parameter. This leaves one optimal model for each of the above data combinations. We calculated goodness of fit *r*^2^ on the test dataset. From this procedure, we selected our reference model with the highest OOS *r*^2^.

This model is used to evaluate overall *r*^2^ results and statistical significances. However, to report the brain properties relevant to our discussion, we increased the regularization parameter by roughly a factor of two from 0.05 to 0.095. Increasing this up to 0.1 constrains the number of variables substantially, therefore we retained this value at 0.095. The OOS goodness of fit *r*^2^ goes down by 5% only (from *r*^2^ = 0.317 ± 0 .017 to *r*^2^ = 0.305 ± 0.021), while using just 1/10th of the optimal number of features (~40 instead of ~400). Given this evidence, we based our feature interpretation on these more-regularized models.

To avoid over-fitting to train-test splits and other chance-events like cross-validation selections, which have a big effect on outcomes, an ensemble of models is obtained by running this model selection procedure 100 times. This entails splitting the data into train and test samples and scoring results using cross-validation multiple times. Train-test splits had a non-negligible impact on results, but performing all data splitting multiple times averages out these effects.

Note that obtaining finely tuned results from specific train-test splits is a common source of confirmation bias (Klayman and Ha, 1987; Klayman, 1995). With our ensemble approach we avoid this pitfall at the expense of having to deal with a multitude of models. This drawback, however, enables us to obtain a robust feature set: We only described variables that are contained in more than 80% of the model ensemble.

The HCP connectivity data integrates numbered ICs. To identify the large brain networks from these ICs, we used a template matching procedure using the canonical network templates (Shirer et al., 2012; Altmann et al., 2015). Each IC was correlated with all canonical network templates and the largest correlation determined the mapping (see Supplementary Figure 6). Individual ICA templates were created using voxels different from 0 and with z score above the 99 percentiles. For visualization, we used the BrainNet viewer (Xia et al., 2013).

To further characterize and interpret these results we used Neurosynth (Yarkoni et al., 2011; Gorgolewski et al., 2015) to decode brain functional activity related to each one of these individual ICA network maps.

Neurosynth is large-scale database of functional neuroimaging data which can map any brain connectivity image to its meta-analysis database. Neurosynth uses a whole-brain reverse-inference to associate specific terms with brain maps. This feature (“decoder”) allowed us to extract words and properties frequently associated with brain areas covered by the HCP ICs. We created word clouds of the most correlated words while filtering out brain morphology descriptors (Supplementary Figure 7). Separate ICs describe different topics, and as expected, ICs classified as equal canonical networks shared similar topics.

### Heritability

The HCP 1200 data also contains information on family structure within the subjects. This information was used to identify subjects in 4 categories: monozygotic (MZ) twins, dizygotic (DZ) twins, full siblings (not twins), and half siblings. Using this information, we calculated the mean Euclidean distance of pairs of data points within the HCP data in the ability trait space for MZ, DZ, full siblings, half sibling and unrelated participants (Figure 4A).

To estimate the heritability of ability traits, we followed the ACE model (Everitt, 2005): To compute heritability components, we compared regression coefficients of within-MZ to within-DZ data using Falconer's formula (Falconer, 1960). Our dataset is small, so we used this simpler approach rather than structural equation modeling which is used elsewhere in the literature (Lynch and Walsh, 1998; Hill and Mackay, 2004; Visscher et al., 2008; Boker et al., 2011).

Following the ACE model, it was possible to estimate the relative contribution of the additive genetic variance (A), common environmental variance (C), and idiosyncratic environment variance (E) for each ability trait. Falconer's formula assumes that MZ twins share 100% of their genetic and shared environmental components, therefore $r_{MZ}^{2}=\;A+C$, whereas DZ twins share 50% of their genetic and 100% of their shared environmental components: $r_{DZ}^{2}\;=\frac{1}{2}A+C$. We also have *A* + *C* + *E* = 1. Here, *r*^2^ are the correlation coefficients for linear regressions of any variable spanned by the twin-pair space.

Note that these assumptions are fairly restrictive compared to structural equation modeling approaches. For example, genetic-environment interactions (Purcell, 2002; Caspi and Moffitt, 2006) are not being modeled here.

These three assumptions can be used to calculate the contributions of the three components from the two regression coefficients for MZ and DZ pairs. In the literature, this genetic component is known as the broad sense heritability *H*^2^. We calculated this measure for the components from the dimensionality reduction in addition to the original variables. Hereditability is reported only for variables and ability traits where the regressions for both, MZ and DZ twin pairs, were statistically significant (*p* < 0.05) (Table 1).

**Table 1:** Heritability estimates of ability traits.

		**Broad sense heritability**	**Shared environment**	**Non-shared environment**	**MZ *p*-value**	**DZ *p*-value**
**Ability traits by gender**						
Motor-endurance	M	0.32 ± 0.23	0.34 ± 0.14	0.34 ± 0.10	<0.001	<0.05
Motor-endurance	F	0.30 ± 0.27	0.35 ± 0.23	0.35 ± 0.09	<0.0001	<0.05
Executive and cognitive function	F	0.22 ± 0.20	0.24 ± 0.28	0.54 ± 0.09	<0.05	<0.05
**Gender corrected ability traits**						
Motor-endurance		0.31 ± 0.22	0.34 ± 0.19	0.35 ± 0.07	<0.0001	<0.001
Executive and cognitive function		0.49 ± 0.22	0.05 ± 0.20	0.46 ± 0.07	<0.0001	<0.05
**Individual ability variable**						
Upper extremity strength—ND-hand	M	0.14 ± 0.34	0.50 ± 0.30	0.36 ± 0.12	<0.001	<0.05
Endurance-−2-min walk—distance	F	0.29 ± 0.43	0.33 ± 0.22	0.38 ± 0.09	<0.0001	<0.05
List sorting	F	0.27 ± 0.17	0.47 ± 0.14	0.25 ± 0.06	<0.0001	<0.001
Picture sequence memory	F	0.37 ± 0.26	0.16 ± 0.22	0.47 ± 0.08	<0.001	0.05

*M, male; F, female; ND, Non-Dominate; MZ, monozygotic twins; DZ, dizygotic twins*.

*Only items for which we have significant correlations for mono- and dizygotic twins were kept. The individual ability variables showed which tasks drove the significant results in the ability traits. Ability trait 1 (Motor-endurance) was driven by Motor-endurance tests, while ability trait 3 (Executive and cognitive function) had strong influences from list sorting and memory tasks. The filtered HCP data includes 52 male and 96 female MZ twins and 38 male and 66 female DZ twins. P-values are calculated comparing traits between individual twin pairs. Confidence intervals include one standard deviation*.

Gender-corrected results were obtained by linearly regressing each variable against a binary gender category and subtracting this result. Using such regression residuals, we can recalculate heritability without dividing the sample into males and females.

## Results

### Data Filtering and Study Design

**Figure 1:**
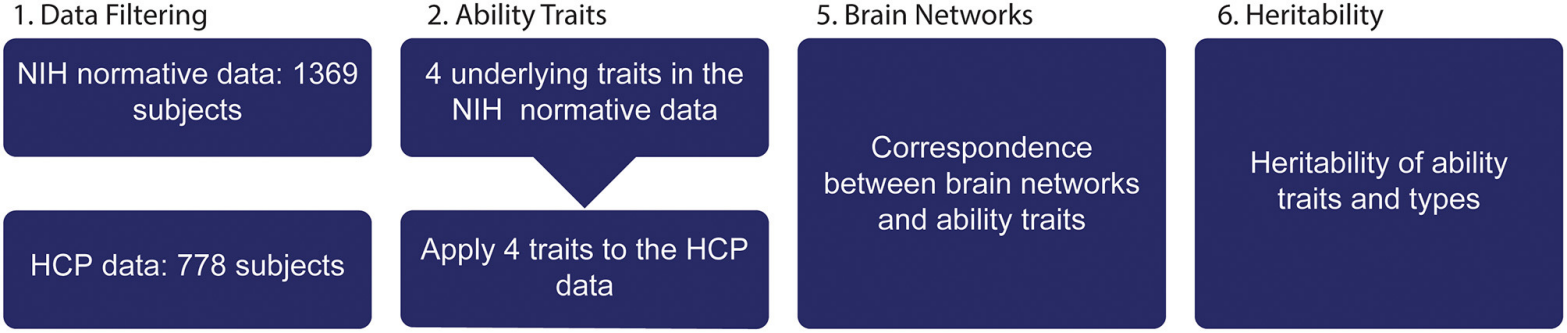
Structure of paper. (1) NIH toolbox data from Normative study and HCP was filtered, only ability data were included in the initial analysis. (2) A dimensionality reduction of the 31 variables was performed exposing four ability traits. (3) The HCP dataset is used to link brain features to ability traits and (4) to determine heritability of individual ability traits and brain networks.

We used data from the NIH normative study (4,852 individuals and 172 variables) (Gershon, 2016) and the HCP S1200 release (1,206 individuals and 261 variables) (Van Essen et al., 2012). Data filtering was performed prior to initiation of data analyses: Only the 31 NIH Toolbox scores present in both datasets were kept (Figure 1 and Supplementary Figure 1), while some remaining variables were later used as covariates. We only included adult subject (18–85 years old, excluding 3,495), who completed all NIH Toolbox assessments, with no more than 30% missing data, excluding 74 participants. Subjects with fMRI quality control issues (*n* = 157) based on notable brain anatomical, processing or data issues (WU-Minn, 2017) were excluded. After data filtering, 1,369 subjects were kept in the NIH normative data and 778 subjects in the HCP dataset. Missing data were replaced by mean values because the overall number of missing data were low (2.7%) and the NIH Toolbox consortium recommends mean imputation (Slotkin et al., 2012).

### Ability Traits

**Figure 2:**
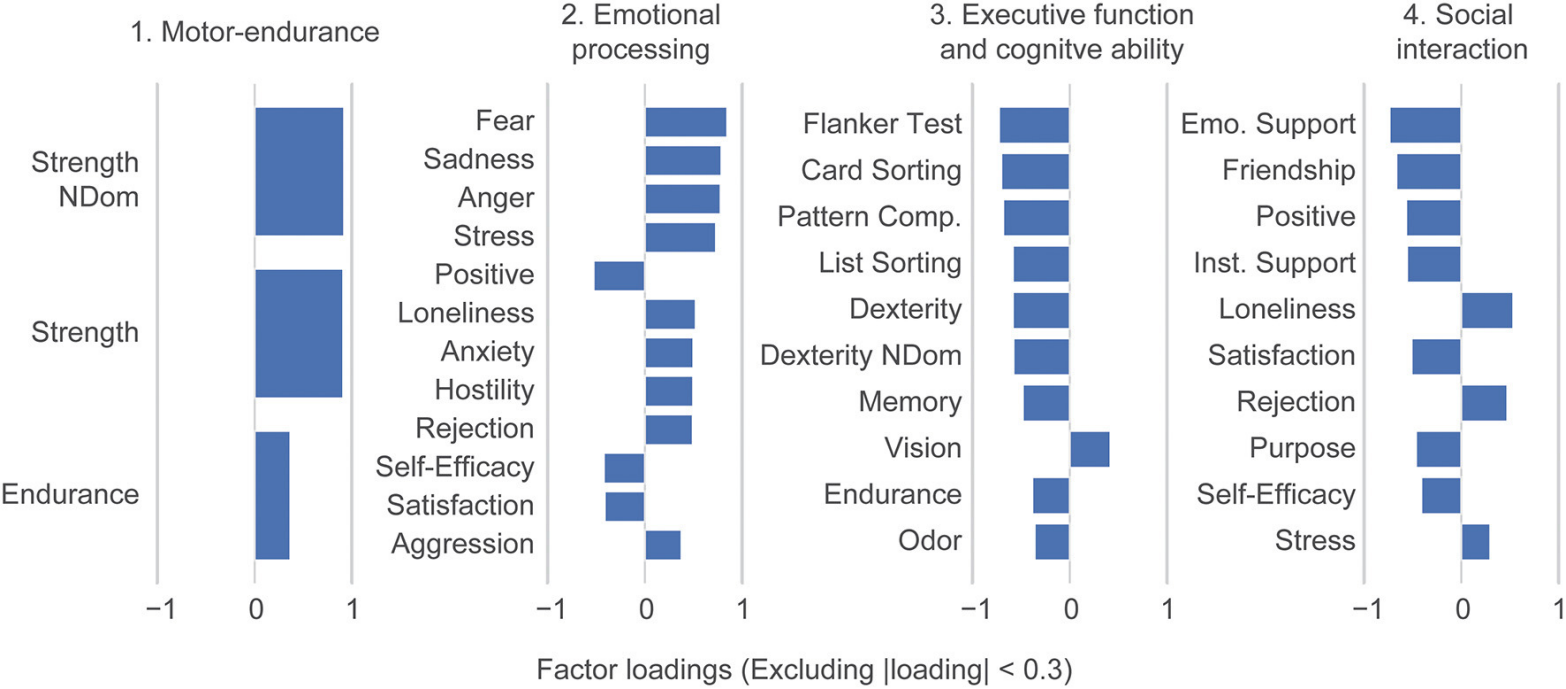
Dimensionality reduction reveals ability traits. Factor loadings in terms of the individual NIH toolbox variables. A factor analysis performed on the 31 individual NIH toolbox variables identified 4 varimax rotated ability traits: (1) Motor-endurance, (2) emotional processing, (3) executive and cognitive function, and (4) social interaction. Loadings smaller than 0.3 are suppressed in the figure. Abbreviations are mapped to NIH toolbox variables in Supplementary Table 4. The dexterity variables are multiplied by negative one because the original value represents the time to test completion, so greater values indicate worse performance. However, all other variables follow the convention that greater values represent better test performance.

Here, we used the NIH Toolbox assessment battery to span a basis space for human ability. To extract underlying ability traits, we performed a factor analysis dimensionality reduction. This defined ability traits as combinations of the original toolbox tasks; we did not include any demographics characteristics in this step of the analysis. Subsequently, four underlying ability traits explained all 31 NIH Toolbox variables up to intrinsic noise (Supplementary Figure 2B) which explains 87.6% of the total variance. Ability traits were interpreted based on their factors loadings as: (1) Motor-endurance, (2) Emotional processing, (3) Executive and cognitive function, and (4) Social interaction (Figure 2). Note that our trait loadings are rotated relative to the canonical toolbox domains of cognition, emotion, sensory-, and motor function.

To further evaluate these properties, we investigated whether brain properties (morphology and connectivity) provided by the HCP dataset arbitrate the ability traits described here.

### Linking the HCP Data

HCP data were used to infer brain properties and degrees of heritability of the four ability traits. To show that both datasets—NIH normative and HCP—are governed by the same underlying traits, clustering analysis was performed on them. The latter identifies groups of subjects with shared factor loadings. Covariates like age, gender, socioeconomic status, and education are then compared across clusters. Equivalence therein shows how meaningful the four ability traits are because they generalize across datasets with different demographics.

The clustering models are trained on the four ability traits of the normative data only, since the traits also exclusively stem from this data. The statistical models used were not affected by the HCP data at all, and only reflect patterns encountered in the NIH normative data. Once the HCP data has been clustered with this model, covariates can be compared.

Both, the Bayesian Information Criterion (BIC), and the Adjusted Rand Index (ARI) favor 4 clusters of participants in the normative data (Supplementary Figure 3A). The BIC compares the accuracy of statistical models to their complexity. The ARI evaluates agreements of subsets of the partitions produced by the clustering model. Robustness was confirmed by applying the same clustering method to different subsets of the data and correspondingly calculated the BIC and ARI scores.

The clusters generalize across both datasets: Although the HCP dataset contains data on younger subjects (from 18 to 35 years old) (Supplementary Figure 5A), the agreement between these clusters with regards to age and gender is tight. The distribution of these variables is consistent along all clusters and reflects the underlying nature of their composition. For instance, cluster 1 is represented mostly by male subjects, clusters 2 and 3 are more dominantly female in both datasets, whereas cluster 4 correlates strongly with increasing age (Supplementary Figure 5B) and is therefore under-represented in the HCP data.

HCP clusters are also compared to a subset of NIH normative subjects from the same age group. This shows that the lower proportion of subjects in cluster 4 and the overrepresentation of cluster 1 in the HCP dataset is in fact a function of age and gender: The young adults in the NIH normative data follow the same patterns as in the HCP but total numbers are re-distributed between clusters 1 and 4. This deviation is caused by the gender ratios within these datasets: The HCP data presents a much lower gender representation at 1.24 females per male compared to the NIH normative dataset at 1.78. The ratios of subjects in the HCP dataset are similar in their first two age bins of the NIH data (Supplementary Figure 5B shows this in detail). Accounting for the differences in gender and age we showed that the ability traits generalize. Note the strong gender and age dependencies in clusters 1 and 4: Gender and age were excluded from the initial data analysis, nevertheless this uncorrected data reveals a strong clustering thereof.

Additionally, the four ability traits explain a similar amount of data variance (81% in the HCP dataset vs. 87% for the NIH normative data), and both datasets favor four underlying ability traits (see Supplementary Figure 2). Therefore, these ability traits reveal adequate convergent and discriminant validity.

### Brain Morphology and Connectivity

So far, we have focused on describing human ability, root causes for these differences stay elusive. To what degree can brain properties predict these ability outcomes? Using morphological data like tissue volume and thickness in conjunction with average functional connectivity strengths between large-scale networks, we assessed the relationship between these biological properties and human ability. Starting out, we regressed brain properties against the four ability traits.

Brain network connectivities were taken from the ICA provided by the HCP (Van Essen et al., 2012). 15^2^-300^2^ features—corresponding to 15–300 independent components—are provided. The HCP FreeSurfer (Fischl, 2012) data contain about 200 morphological variables.

Functional connectivities with 50 independent components performed best in the brain properties vs. traits regressions. For the brain connectivity features, the HCP's more strongly regularized network matrices dataset of functional connections (netmats2) are favored. Netmats2 aims to estimate direct connection strengths better than netmats1 (see section Methods: Brain Morphology and Connectivity). The model favored by the data includes the gender, morphological and connectivity variables with a total of about 400 features. However, since we were primarily interested in model interpretation, we reduced the complexity of this model by doubling its regularization coefficient from its optimal value (details in section Methods: Brain Morphology and Connectivity). This leaves us with 40 features without severely reducing goodness-of-fit.

Interestingly, final out-of-sample (OOS) model results depend more significantly on the train-test splits than the exact choice of independent variables. The difference between netmats1 and 2, for example, is small. To address this issue, we performed this entire fitting method 100 times to get statistically significant results. The confidence intervals around below results, for example, are obtained this way. All quoted numbers are averages across this ensemble of 100 models.

The independent components for ability trait 1 (Motor-endurance) and 3 (Executive and cognitive function) contain 73 and 91% connectivity variables, respectively, and also include gender as a predictor. The rest of the model ensemble is made up by morphological features. We fitted against all four ability traits, for which the OOS *r*^2^ = 0.302 ± 0.017 (adjusted *r*^2^ = 0.222 ± 0.023). Individually, trait 1 has an average goodness of fit *r*^2^ = 0.78 and *p*-value close to 0 and trait 3 has *r*^2^ = 0.27 and *p* = 0.003 (all OOS). *P*-values are calculated regressing predicted and real ability trait values.

Ability traits 2 and 4 are not statistically significant with average OOS *p*-values of 0.1 and 0.4, respectively. These are the ability traits dominated by emotional and social interaction variables, and, apparently, are less robustly reflected in the brain morphology or functional connectivity.

The best statistical model includes gender as a feature. When comparing model results with and without gender, *r*^2^ drops by 50%. Specifically, ability trait 1 predictions are dominated by gender, which is unsurprising, because this describes Motor-endurance which is strongly gender dependent.

**Figure 3:**
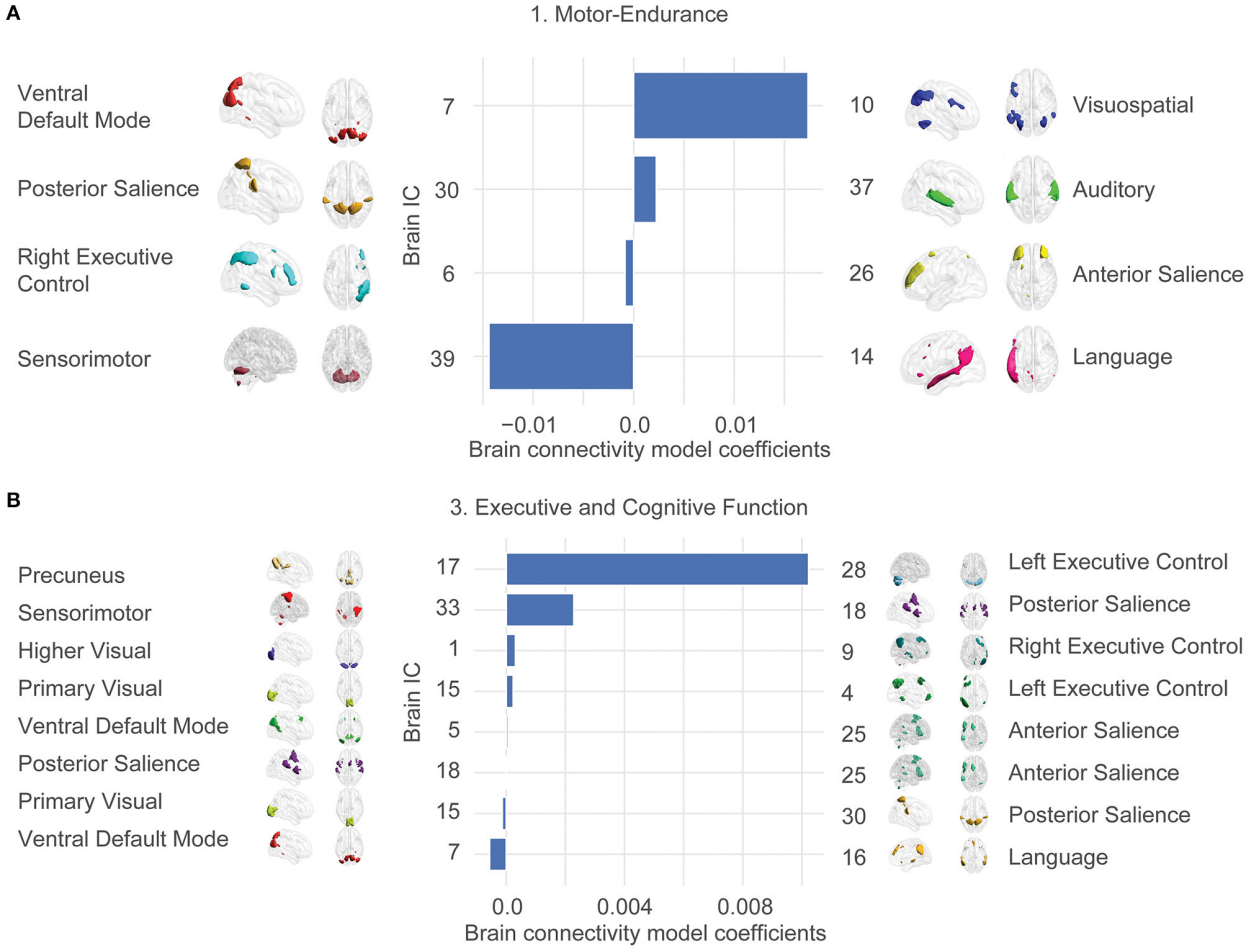
Large-scale network connectivity determines ability trait 1 (Motor-endurance) and 3 (Executive and cognitive function). The large-scale network links were determined by averaging more regularized models and displaying the average coefficient for features that are prevalent in each model. Prevalence is determined by presence in 80% of the models. Only the two significant ability traits are displayed. Ability trait 2 and 4 show insignificant predictions out of sample. Template matching using canonical network templates (Shirer et al., 2012; Altmann et al., 2015) was performed. For visualization, ICA maps derived from the HCP consortium (dimensionality = 50) were thresholded at the top 1% *z*-score for non-null voxels and brains were displayed using the BrainNet viewer (Xia et al., 2013). Note that the independent variables are all z-scored, so parameter values can be compared. The bars represent average model coefficients, not connectivity values. **(A)** Ability trait 1 (Motor-endurance) connectivity model coefficients. More connectivity between IC 7 and 10 indicates higher values of trait 1, for example. **(B)** Ability trait 3 (Executive and cognitive function) brain connectivity model coefficients.

We were interested in the exact anatomy that provides these relationships. Figure 3 shows the most significant brain features involved in our final model. This plot does not contain any morphological features since they overfit to the exact train-test splits and are not reliably selected.

In contrast, the statistically significant associations link ability traits 1 (Motor-endurance) and 3 (Executive and cognitive function) to correlations across a series of networks including executive control, the default mode, salience, precuneus, sensory-motor, language, auditory, and visual networks. Independent components were correlated against an atlas of regions of interest for interpretability.

The coefficients of these large-scale network links and their contribution to the individual ability traits are displayed in Figure 3. There were stronger positive associations between the Motor-endurance ability trait and the inter-network connectivity of the auditory and the posterior salience network and the visuo-spatial and ventral default mode network connectivity. In contrast, there is a negative relationship to the sensorimotor-language connectivity, representing coordination and speech production (Figure 3A). These relate to connectivity patterns that have previously been implicated in sensory and motor skills such as visuospatial orientation, attention, and tactile sensations (Johansen-Berg et al., 2002; Erickson et al., 2015).

Regarding Executive and cognitive function, the most prominent feature integrates the left executive control with the precuneus network and the posterior salience with the sensorimotor network. These are all positively correlated to the executive function ability trait (Figure 3B). On the other hand, language and ventral default mode networks presented stronger negative coefficients. This pattern involving negative integration of motor/visuospatial networks was previously associated with lower working memory in healthy subjects and in schizophrenia and major depression disorder. Yamashita et al. (2018) showed that working memory was most strongly related to within-network functional connections of the left fronto-parietal network, contributing 1/3 of the total variance. The next biggest contributions at ~1/4 of the total variance each come from connections between the supplemental motor and the primary sensorimotor networks as well as the cingulo-opercular network connected to the midbrain, in accordance with what we obtained.

Overall, we showed that two ability traits (Motor-endurance and Executive and cognitive function) were reliably associated with functional connectivity between brain networks consistent with, and specifying, previous literature results. Next, we investigated whether heritability plays a role determining the ability traits.

### Heritability of Ability Traits

Human ability changes over time, however, with cross-sectional studies like these, such effects cannot be captured. Nonetheless, more static ability can be observed using the heritability of ability traits. Here, we used the restricted HCP data containing information on family structure within the participants to identify genetic influences among the ability traits. In our cleaned HCP dataset (*n* = 714 after excluding subjects with unclear family structures or no genotyped results) we identified 148 subjects that are monozygotic (MZ) twins, 104 dizygotic (DZ) twins, 548 subjects that are full siblings and not twins and 25 subjects who are half siblings. The sum of these numbers is >714 because participants can be both twins and half-siblings, for example, so they can appear more than once.

**Figure 4:**
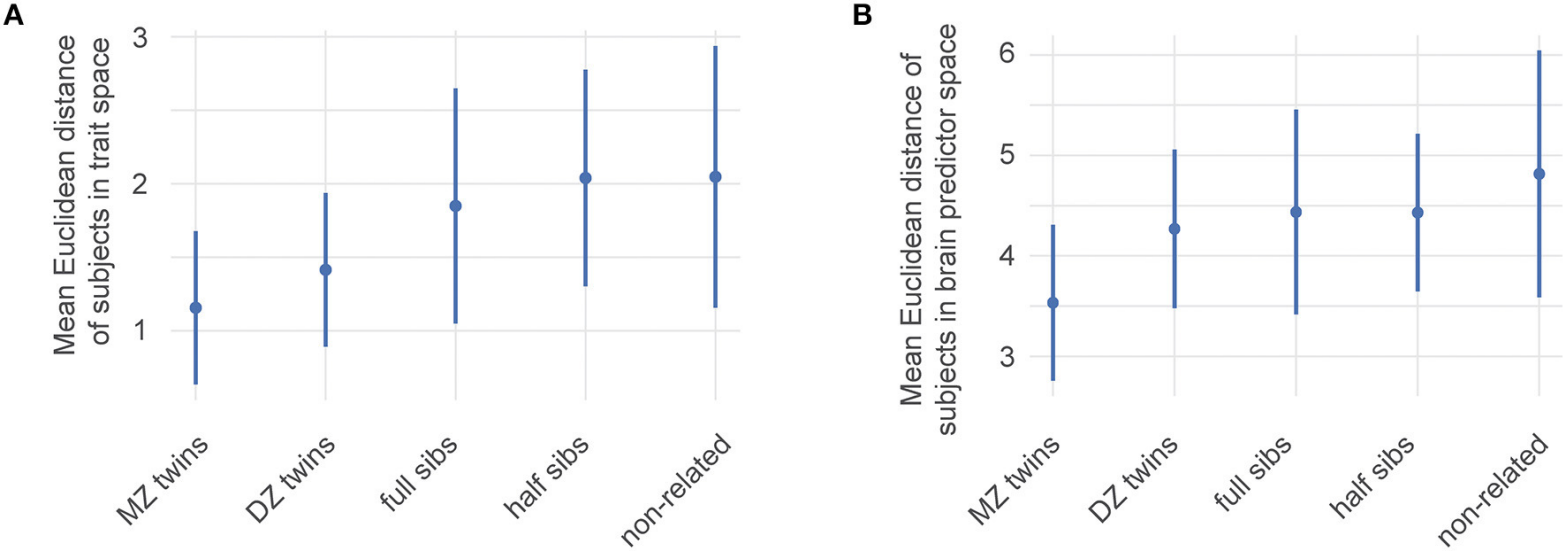
Estimates of the genetic influence on ability traits. HCP family structure data were used to identify genetic influences among the ability traits and brain networks contributing to them. Mean Euclidean distance of pairs of data points within the HCP data. MZ: monozygotic twins, DZ: dizygotic twins Error bars correspond to one standard deviation of distances of the corresponding subset. **(A)** Distances between pairs of subjects within the four-dimensional ability trait space. **(B)** Distances between subjects using the space spanned by the 19 relevant features from Figure 3. A higher genetic overlap leads to smaller distances.

We first characterized genetic influence along the ability traits by simply calculating the Euclidean distance between twins, siblings, and unrelated subjects in ability trait space (Figure 4A). This distance grows with a decrease in the genetic overlap between siblings which suggests a genetic influence over the abilities we identified.

Having shown this clear trend, we calculated numerical heritability indexes of the ability traits. These calculations assume that every ability trait can be explained by a combination of genetic, shared environmental, or non-shared environmental factors. These three type contributors can be obtained by comparing overlaps between MZ twins and DZ twins, because both presumably share all environmental factors, and a well-defined fraction of genetic ones. For this analysis, genders are separated because for DZ twins of opposite sex these assumptions no longer hold. Table 1 shows results for this analysis.

Ability traits 1 and 3 (Motor-endurance and Executive and cognitive function) presented significant results of around 30% broad sense heritability for ability trait 1 (females and males), and 22% for ability trait 3 (females only). In contrast, the emotional traits showed no significant heritability. To potentially uncover additional dependencies, and in spite of the importance of gender in the heritability index calculation and the overall role of this variable in the definition of the ability traits, we additionally performed the same analysis on gender-corrected data, ignoring theoretical assumptions. The results obtained, however, are very similar results to the above, in that ability traits 2 and 4 remained insignificant (Table 1).

Similarly, we also calculated the gender-dependent heritability for each original test variable. The results show which underlying ability traits are driving the findings in Table 1. Note that some ability traits—self-efficacy, pattern comparison score and the upper extremity strength—have negative heritability coefficients. In the literature, these are treated as a breakdown of the model (Burton et al., 2018), and are excluded from results.

Similar results have also been shown for general ability and personality traits in the literature (Robinson et al., 1992; Vukasović and Bratko, 2015), showing that both Motor-endurance and Executive and cognitive function have genetically influenced components. Note that ability traits 1 and 3 are significant both in the brain predictions, and for the broad sense heritability.

Our selection of predictive brain features allows us to compare how individual sibling types differ from each other in their brain connectivity. To illustrate this, we focused on their distances in the space spanned by the 19 prevalent features describing the Motor-endurance and Executive and cognitive function ability traits. This is equivalent to the above illustration in ability trait space, unsurprisingly, Figure 4B shows that large brain networks are more similar in siblings with more genetic overlap.

Overall, these heritability results link nicely with the significant factors in the twin analysis (Table 1) and point out the biological, as opposed to behavioral, root causes describing ability traits 1 and 3.

## Discussion

In this paper we used the NIH Toolbox individual measurements (31 scores) to identify ability traits in healthy subjects and associated these properties with functional brain connectivity and genetic disposition. We identified four ability traits that can consistently describe the participants. Two of these ability traits, Motor-endurance and Executive and cognitive function, were associated with connectivity between large brain networks and influenced by genetic disposition. Regarding the ability traits, the first (Motor-endurance), reflects increased physical fitness and endurance of participants; the second and fourth ability traits (2: emotional processing and 4: social interaction) reflect social and emotional properties, including psychological well-being, stress, negative affect and social purpose. The third ability trait (Executive and cognitive function) shows processing speeds, attention, episodic, and working memory (see Figure 2). The data used in this paper are cross-sectional and ability traits are comprised of outcomes that can change over subject's lives. Therefore, age and gander variables were not included in the factor analysis; even though we expect changes in behavior over time in addition to gender differences. Additionally, excluding age and gender focuses more purely on ability. For example, in Supplementary Figure 5, using the clusters, we show that the executive function trait, which anti-correlates strongly with age, still captures ability independent thereof.

We used brain morphology and connectivity to characterize the networks involved with the ability traits and identified whether these were hereditary. It is noteworthy to point out that these two approaches differ significantly from each other. One selects from brain features that relate closely to the identified ability traits. The other one compares how similar different kind of twins respond to surveys and perform in tasks. Defying those unequal analyses, the results equivalently point at biological root causes of human ability: Ability traits 1 and 3 (Motor-endurance and Executive and cognitive function) are predictable by the connectivity between large brain networks and are both significantly hereditary.

The significant functional connections involved in the characterization of ability traits 1 and 3 comprise a series of brain networks including the default mode and salience network and their associations with sensory-motor, language, auditory, and visual networks as part of the ventral attention network. With respect to the ability trait 1, a recent study also showed that alterations in the inter and intra connectivity of the default-mode-network (DMN) with dorsal attention, somatomotor, salience, and executive control networks can explain the variance in cardiorespiratory fitness independent of physical activity (Voss et al., 2016). Not surprisingly, the relationship between physical fitness and cognition is also consistent with previous studies showing that functional DMN connectivity mediates this relationship (Voss et al., 2010). Numerous studies show that at rest, both the salience and attention networks (i.e., visuospatial, perception) coordinate the processing of information by regulating the DMN activity (Chen et al., 2013; Cohen and D'Esposito, 2016). The dynamical connectivity between and within these areas is related with variability in the performances in cognitive tasks, such as working memory tasks. They also identified similar large-scale network connectivity that correlated with working memory performance in neuropsychiatric conditions such as schizophrenia, major depressive disorder, obsessive compulsive disorder, and attention deficit disorders (Yamashita et al., 2018). Altered connections in the executive control networks were observed across the four conditions. Besides that, visual network alterations were also associated with lower working memory ability. Taken together, our results show that functional connections of brain networks associated with the Executive and cognitive function ability trait were consistent with the literature, while being more specific. Task control networks associated with Executive and cognitive function in Figure 3B are known to exert top-down regulation of sensorimotor processing as well as interact with DMN influencing behavioral performance (Wen et al., 2013).

The emotional and social traits were not reliably associated with brain connectivity or morphology. We caution against concluding that there are no such dependencies. While morphological changes require more chronic, pathological dispositions, resting state connectivity has been shown to correlate with emotional traits (Roy et al., 2009; Blackford et al., 2014; Guell et al., 2018). These studies, however, focus on individual brain features (amygdala, pars triangularis, superior temporal gyrus among others) or even subsets thereof, whereas here, more broad IC maps are being used. It is in this context that more fine-grained networks are ignored and thus do not reliably relate to emotional and social traits. Future studies could address this question by selecting from larger ICA decompositions, like 300 instead of our 50, which reveals smaller networks. Here, however, since more ICs overfit overall results, such detailed predictors remain hidden.

Here, we only evaluated relationships between large networks at rest with variables from the NIH Toolbox assessment battery. However, it is important to point out that the activity of these brain networks and subnetworks also correspond to resting and task-related connectivity patterns (Smith et al., 2009). Our results support that increased performance in a specific ability trait may depend on the intrinsic correlation between these pairs of networks.

In addition to the characterization of the networks involved with the ability traits, we investigated the effects of genetic influence on traits. Behavioral and ability traits are both sensitive to environmental influences. However, we showed that ability traits 1 and 3 (Motor-endurance and Executive and cognitive function) were also strongly influenced by genetic disposition. Broad sense heritability accounts for about 31 and 49% of the observed variance in each ability trait, respectively. Given the associations observed by us between the Motor-endurance ability trait and functional connectivity, the genetic contribution is in line with previous reports of strong genetic influence (about 50%) over the cardiorespiratory fitness and how it accounts for the specific association between cognition and physical fitness (Bouchard et al., 2011). Therefore, individuals that are genetically predisposed to higher levels of cardiorespiratory fitness would present better performance in tasks related to the Motor-endurance ability trait independent of age. Additionally, it is possible that they experience the most protection against adverse effects of aging on the brain—which ultimately has important clinical implications.

Our findings with respect to the heritability of the Executive and cognitive function ability trait is also well-substantiated, since previous studies showed a moderate heritability around 30% of working memory in different twin population samples (Singer et al., 2006; Zhou et al., 2018). While Executive and cognitive function heritability shows much more varying results in the literature—estimates range from 27% to about 77%, it is important to point out that the properties of the Executive and cognitive function ability trait are much more complex than only working memory or cognition alone and the strong genetic influences observed by us can be a result of additive genetic factors within attention, episodic memory, and cognition.

Moreover, our results are also corroborated by a recent publication computing heritability across the HCP dataset using the NIH toolbox domains (Christova et al., 2020). Although we apply factor analysis to obtain four traits, they can be mapped into the four NIH Toolbox domains of motor, cognition, emotion and sensory. Similar to our results, Christova et al. showed that the heritability estimates for the NIH Toolbox motor domain ranges from 13 to 29%—compared to 30% in our motor-endurance trait—and from 36 to 48% in the cognition domain—compared with 22–40% in the Executive and cognitive function trait. Moreover, Christova et al. showed that the heritability of the NIH Toolbox emotional domain is about 35%, which we were not able to confirm. To compute heritability, we only considered significant inter-twin correlations (*p* < 0.05), which the emotional processing and social interaction traits did not show. Note, however, that our traits, as mentioned above, are not identical to the emotional NIH Toolbox domain.

In summary, both the Motor-endurance and the Executive and cognitive function ability traits showed significant heritability factors. From a top-down point of view this result is not surprising because both point to common genetic factors that determine these outcomes.

We also showed that common genetic components are partially responsible for individual variabilities in network connectivity, by showing that the 19 large brain networks included in our model (related to traits 1 and 3) are progressively more similar in MZ twins than DZ twins and siblings (see Figure 4B). Similarity in functional connectivity between these large brain networks is therefore proportional to the shared genetic background. A recent study, using the HCP functional magnetic resonance imaging (fMRI) data, estimated the heritability of 39 cortical regions, and showed that on average, broad sense heritability accounted for about 15% of the observed variance in fMRI connectivity (Colclough et al., 2017). Similar results connecting heritability and Executive and cognitive function and motor skills have been found in Bouchard et al. (1997), Beunen et al. (2003), Damoiseaux et al. (2006), and Heutink et al. (2006). Generally, there are strong results between heritability and network connectivity (Smit et al., 2008; Jansen et al., 2015; Yang et al., 2016; Colclough et al., 2017).

A recurring theme in this analysis is the sparsity of data regarding certain measures. Even though the HCP dataset is large compared to other fMRI collections, its analytic power in the heritability and brain feature regime is limited by its size. Both, gender-independent ability trait predictions as well as more significant heritability results require larger datasets. Specifically, twin studies are often one or two orders of magnitude larger (Lichtenstein et al., 2002; Trouton et al., 2002; Moayyeri et al., 2013). However, their scope often is much narrower. Direct classifications based on brain features would only be possible with larger datasets. Some of the open questions following this paper will therefore easily be addressed once larger datasets have been acquired.

On the other hand, studying brain-ability connections and corresponding heritability analyses have only been made possible by providing a standardized framework, in this case the NIH Toolbox. This points out the power thereof: Once a common denominator has been established, many pivoting analyses can be performed from there. This enables a large degree of generalizability of research. Including NIH Toolbox surveys in other data acquisition projects would allow for extensions of this analysis to other fields.

In this paper, we found a characterization of human ability that is complete with respect to the NIH Toolbox tasks, and succinctly describes them with just four ability traits. We quantified which ability traits are rooted in brain networks and found to which degree these ability traits are genetically influenced. Looking forward, this framework allows us to determine connections between the trait space and clinical outcomes.

## Data Availability Statement

Publicly available datasets were analyzed in this study. This data can be found here: https://www.healthmeasures.net/explore-measurement-systems/nih-toolbox; http://www.humanconnectomeproject.org/data/.

## Author Contributions

AA, DR, CP, and JB designed the research. JB and RJ analyzed the data. CP and JB drafted the manuscript. DR, JG, and AA provided critical revisions. All authors contributed to the article and approved the submitted version.

## Conflict of Interest

The authors declare that the research was conducted in the absence of any commercial or financial relationships that could be construed as a potential conflict of interest.

## Footnotes

^1^For PCA and factor analysis we have *A* = *Q* · *P* where *A* is the original data matrix, *Q* are the loadings and *P* maps these onto the original variables. Now *A* = *Q* · *P* = *Q* · 𝕀·*P* = *Q* · (*R*^−1^ · *R*) · *P* = (*Q* · *R*^−1^) · (*R* · *P*) ≡ *Q*′ · *P*′, and *Q*′ and *P*′ are the new loadings and projection matrices. *R* is a rotation matrix.
