# Supplementary material for: Neural and Genetic Bases for Human Ability Traits
Source: Front Hum Neurosci. 2020 Dec 16;14:609170. doi: 10.3389/fnhum.2020.609170 (PMC7772246; doi:10.3389/fnhum.2020.609170)
Supplement: Supplementary file 1 [file Data_Sheet_1.pdf]

Supplemental Material

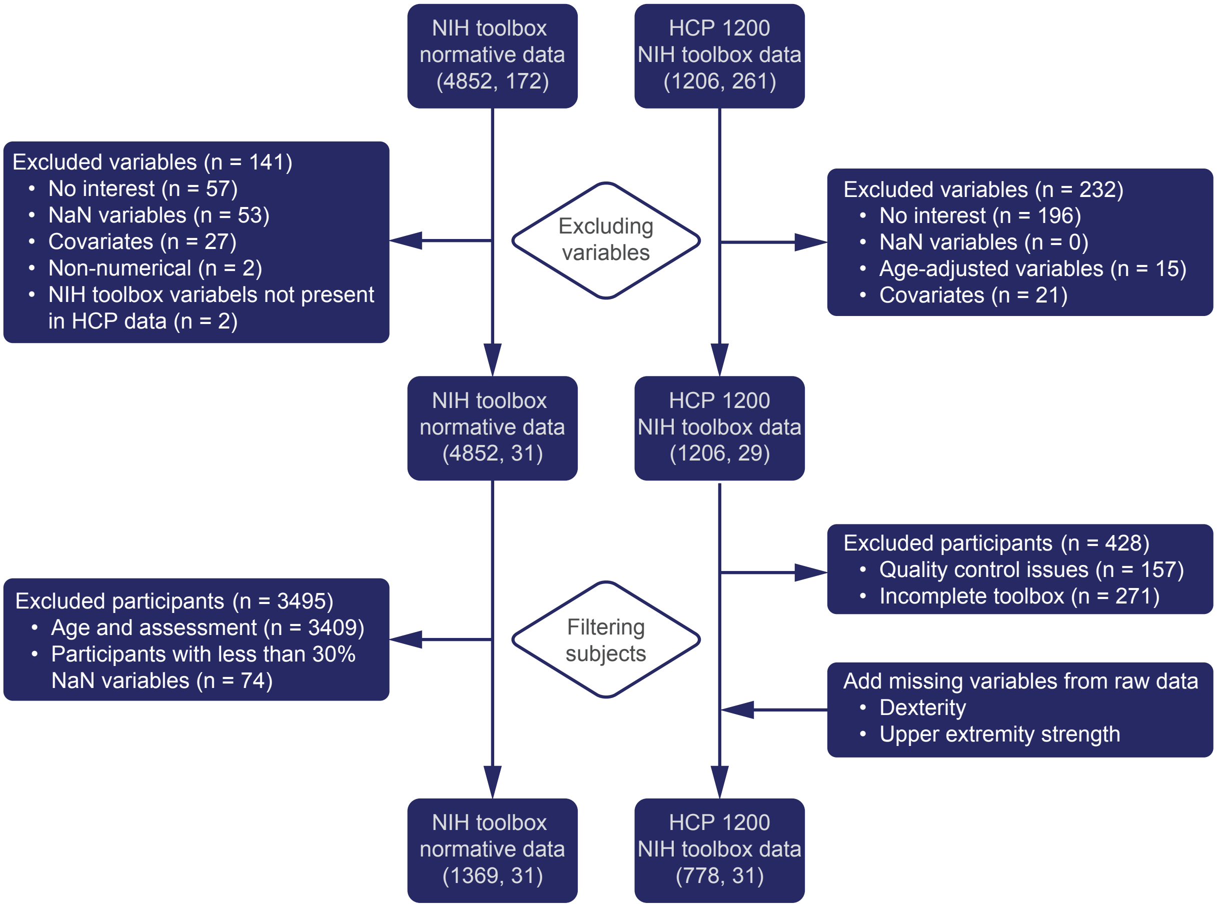

Figure S1 – Data filtering and Variable selection.
This study uses data from the NIH normative study (4852 individuals and 172 variables) and the Human Connectome Project (HCP) S1200 release (1206 individuals and 261 variables). Data filtering was performed prior to any data analysis. Only NIH Toolbox scored variables present in both datasets were included. This analysis was performed age- and gender blind, so we excluded these variables from the fitting dataset. Demographic characteristics were also excluded. Only participants ages 18 and above that reported more than 70% of the variables in question were included and missing data were replaced by mean values.
Left: Filtering the NIH toolbox normative data: 1369 subjects are kept.
Right: HCP data cleaning: 778 subjects are kept. Missing variables from the raw data were upper extremity strength and dexterity. These are only available in the raw and are equivalent to the NIH normative variables. All participants with quality control issues in the MRI were excluded.

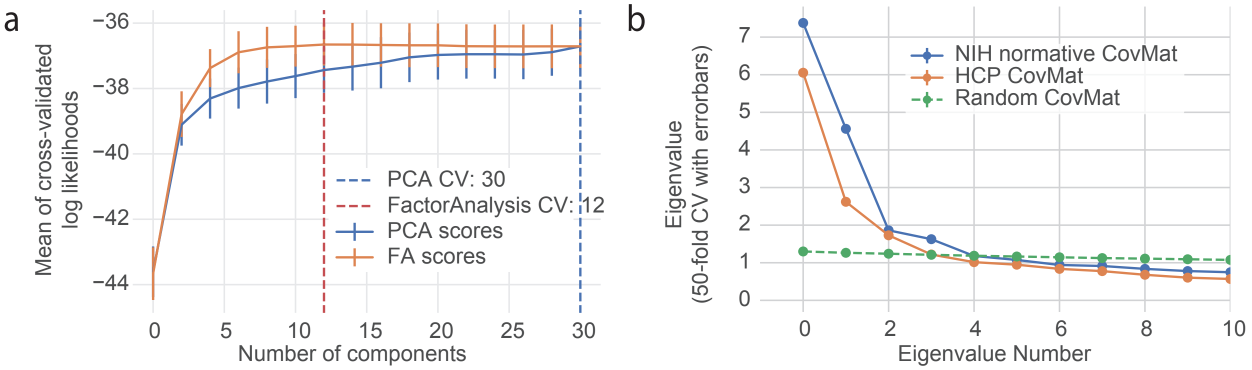

Figure S2 – Dimensional reduction

a: Comparison between factor analysis (FA) versus principal component analysis (PCA). Error bars and mean values are obtained using 5-fold cross validation. Vertical dashed lines show at what point the maximum of each averaged likelihood is reached (12 for FA, 30 for PCA). The better performance of FA over PCA indicates heteroscedastic noise in the NIH toolbox data. Straightforwardly comparing log likelihoods between these two models shows the benefit of using FA – an earlier saturation – earlier maximizing the likelihood – translates to being able to explain more variance with fewer components.

b: Size of covariance matrix eigenvalues: The green dashed line corresponds to a covariance matrix linking 31 standard normal random variables. The blue line corresponds to the NIH normative data, the orange one to HCP data. For both datasets, the fifth eigenvalue crosses the random noise line, hence four ability traits best explain the data while simultaneously keeping the noise level low. Error bars in b are included but are smaller than the dots.

Cluster Analysis

The cluster analysis results used to study the traits to merge normative with the HCP young adult data will be outlined here. While their utility in tying the datasets together has been shown above, here, the focus is on observations within the clusters themselves.

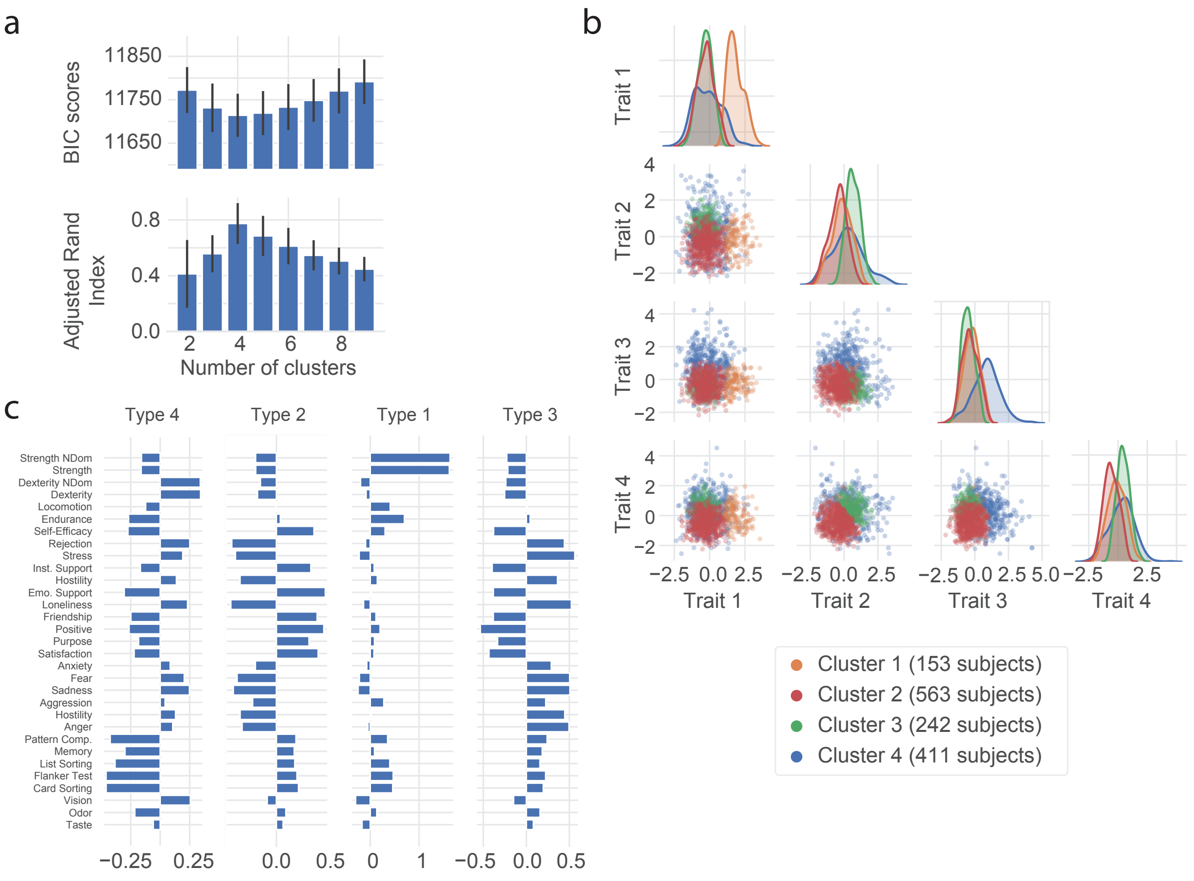

Figure S3 – Optimal number of clusters and their characterization.
a: The top plot shows the BIC scores resulting from the different solutions. The four-cluster solution presented the lowest BIC score, which is backed up by the adjusted rand index in the bottom plot, which also peaks at four. The dataset is sub-sampled 200 times – the BIC score at 80% of its original size, and the Rand index at 60% because this converged faster.
b: Visualization of the data along all ability traits, showing that the clusters are all relatively homogenously distributed; cluster size are also reported. On the diagonal, marginal distributions along each ability trait are displayed, whereas the off-diagonal elements show a scatterplot of the data along each pair of ability traits. Note that the cluster sizes vary.
c: The cluster centers represented in terms of the original variables. This underlines the choice of our cluster IDs. Full variable name available in Table S4.

For easier interpretability the four clusters were identified as: 1) Physical strength 2) Positive emotions, strong executive and cognitive function, high social interaction, low endurance, 3) Negative emotions, low social interaction, and 4) Low executive and cognitive function. These interpretations are also backed up by looking at the original variables directly (Figure S3c). Cluster centers were robust to perturbations in the data: Drawing subsamples from the entire dataset and performing GMMs on them yields equivalent results (Figure S4).

Covariates are used above to show equivalency between traits in both datasets. Tables S1 and S2 summarize these covariates, like socioeconomic status, in detail. These are provided by the

NIH Toolbox and the HCP young adult dataset. In the tables, first, the largest outlier from the mean is determined, and p-values of the distributions are calculated with respect to this outlier. A value of 1 marks the type that provides the outlier. A value less than ~0.01 is considered statistically significant (Bonferroni corrected for multiple comparisons). The tables only indicate the significance of the outliers, not the direction thereof.

Table S1 – Demographic and characteristic differences between ability clusters, NIH normative dataset.
Each covariate is compared to the biggest outlier from the mean of the clusters; for example, the Age (in years): The low executive and cognitive function cluster is the biggest outlier, and it is significantly different from the other three clusters. Displayed are p values comparing to the outlier cluster.

Covariates/Cluster	Cluster 1: Physical strength	Cluster 2: Positive emo., strong exec. Fct., high soc. Interac., low endurance	Cluster 3: Negative emo., low social interac.	Cluster 4: Low executive and cognitive function
		p-values		
Age, years	0	0	0	1
Participant Gender	1	0	0	0
Adult participant's race[1]	0.118	1	0.215	0.498
American Indian or Alaska Native	1	0.005	0.028	0
Asian	0.19	0.223	0.035	1
Black or African American	0.048	1	0.047	0.02
Native Hawaiian or Other Pacific Islander	1	0.23	0.374	0.265
White	0.136	1	0.191	0.304
Ethnicity[2]	1	0.005	0.131	0
Handedness	0.043	1	0.021	0.456
Numeric value for years of education[3]	0	0	0	1
Site	0	0	0.002	1
Language of administration	1	0.005	0.107	0
English Reading, n Correct	0.039	0.004	0.101	1
English Reading, T. Score	0	0	0	1
English Vocabulary T. Score	0	0	0	1
Spanish Reading, n Correct	0.453	0.312	0.663	1
Spanish Reading T. Score	0.004	0	0.002	1
Spanish Vocabulary, n Correct	0.305	0.076	1	0.031
Spanish Vocabulary T. Score	0.056	0	0	1

In the past 7 days how much did pain interfere with your ability to participate in social activities?	0	0	0.341	1
In the past 7 days how much did pain interfere with your day to day activities?	0	0	0.188	1
In the past 7 days how much did pain interfere with your household chores?	0	0	0.275	1
PROMIS-29 Pain Interference: T-Score	0	0	0.366	1
PROMIS-29 Pain Interference: raw sum score	0	0	0.209	1

1: Quantified into numerical values; 2: Like 1, but with regards to Ethnicity; 3: self-report for Adults; N: number; T.: Theta.

Table S2 – Demographic and characteristic differences between ability clusters, HCP dataset. Each covariate is compared to the biggest outlier from the mean of the cluster; for example, the age: The low executive and cognitive function cluster is the biggest outlier, and it is significantly different from the other three clusters. Displayed are p values comparing to the outlier cluster.

Covariates/Cluster	Cluster 1: Physical strength	Cluster 2: Positive emo., strong exec. Fct., high soc. Interac., low endurance	Cluster 3: Negative emo., low social interac.	Cluster 4: Low executive and cognitive function
	p values			
Age, years	0.118	0.024	0.178	1
Gender	1	0	0	0
Race				
Am. Indian/Alaskan Nat.	0.731	0.319	1	0.319
Asian/Nat. Hawaiian/Other Pacifics.	0.244	0.182	0.174	1
Black or African Am.	0.02	0.006	0.095	1
White	0.004	0.001	0.084	1
More than one	0.01	0.054	1	0.004
Unknown or Not Reported	0.801	0.641	0.722	1
Ethnicity	0.132	0.126	0.211	1
Handedness	0.117	1	0.363	0.388
Weight	1	0	0	0
Height	1	0	0	0
BMI	1	0	0.045	0.246
Self-reported BMI category (SSAGA_BMICat)	1	0	0.013	0.081

BMI category for their heaviest period (SSAGA_BMICatHeavies)	1	0	0.002	0.015
years of education completed Years of education (SSAGA_Educ)	0.279	0.027	0.142	1
Employment status (SSAGA_Employ)	0	0	0.002	1
Still in school (SSAGA_InSchool)	0.281	0.203	0.506	1
Total household income (SSAGA_Income)	0.007	0.003	0.694	1

Gender and age are obvious examples. Race and ethnicities are mostly insignificant. Years of education is even across all clusters but the low executive and cognitive function one (~11 vs ~14 years). The language and site of administration follows this trend, although all of these variables are typically strongly correlated and should not be interpreted in isolation. The language reading and vocabulary scores follow the same distributions. Interestingly, the low executive and cognitive function cluster seems to be suffering from higher amounts of pain than clusters 1 and 2.

The weight and BMI of participants in cluster 1 is significantly higher than the remaining three groups. Household incomes in clusters 1 and 2 are also significantly higher compared to cluster 4. Ethnicities and race are distributed evenly across all clusters, whereas education levels are significantly lower in the low executive and cognitive function group.

Eisenberg et al. (Eisenberg et al., 2019) argue that the utility in these clusters lies in real-world comparisons. We show that the structure found in the NIH normative and HCP datasets is valid because it mirrors conditions met outside the data.

Summarizing, these cluster characterizations were equivalent across two separate datasets. Besides gender and age, socioeconomic status, education levels, ethnicity and race distribute similarly across the four cluster in both datasets. Differences in real-world outcomes based on the clusters solidify the usefulness of the traits they are based on.

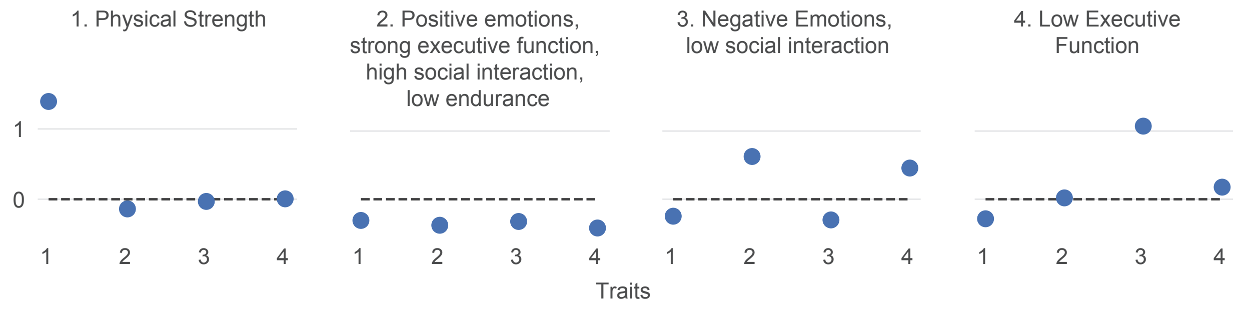

Figure S4 – Clustering reveals types of participants.

Cluster centroids separate the data into four distinct types, as determined by the relative weights of the four ability traits. The four types are labeled as: 1) physical strength; 2) positive emotions, strong executive and cognitive function, high social interaction, and low endurance; 3) negative emotions and low social interaction; 4) low executive and cognitive function. Error bars correspond to re-fits of the GMM on subsampled NIH normative datasets and are too small to be shown. The dashed line at zero shows the sum of each ability trait weighted by the number of participants. The y axis is shared between all four graphs, numbers in the x axis represents the four ability traits identified in Figure 2.

Table S3 – Brain large scale networks independent components.
Brain Networks presented in the final model derived from the independent component analysis provided by the HCP consortium (dimensionality = 50) were characterized using the canonical network templates.

ICA	Canonical Network Templates
1	Higher Visual Network
4	Left Executive Control Network (LECN)
5	Ventral Default Mode Network
6	Right Executive Control Network
7	Ventral Default Mode Network
9	Right Executive Control Network
10	Visuospatial Network
14	Language
15	Primary Visual Network
16	Language
17	Precuneus Network
18	Posterior Salience Network
25	Anterior Salience Network
26	Anterior Salience Network
28	Left Executive Control Network (LECN)
30	Posterior Salience Network
33	Sensorimotor
37	Auditory
39	Sensorimotor

Table S4 – Full names from NIH toolbox individual assessments and the abbreviations used in the manuscripts.

NIH Toolbox Identifier	Abbreviation
Taste Intensity - Whole mouth: Quinine	Taste
Adult Odor ID Score	Odor
Static Visual Acuity logMAR score	Vision
DCCS computed score (0-10)	Card Sorting
Flanker computed score (0-10)	Flanker Test
List Sorting score	List Sorting
Picture Sequence Memory Theta score	Memory
Pattern Comparison Score	Pattern Comp.
Anger-Affect 18-85, theta	Anger
Anger-Hostility 18-85, theta	Hostility
Anger-Physical Aggression 18-85, theta	Aggression
Sadness 18-85, theta	Sadness
Fear-Affect 18-85, theta	Fear
Fear-Somatic Arousal 18-85, theta	Anxiety
General Life Satisfaction 18-85, theta	Satisfaction
Meaning and Purpose 18-85, theta	Purpose
Positive Affect 18-85, theta	Positive
Friendship 18-85, theta	Friendship
Loneliness 18-85, theta	Loneliness
Emotional Support 18-85, theta	Emo. Support
Perceived Hostility 18-85, theta	Hostility
Instrumental Support 18-85, theta	Inst. Support
Perceived Stress 18-85, theta	Stress
Perceived Rejection 18-85, theta	Rejection
Self-Efficacy 18-85, theta	Self-Efficacy
Endurance - 2-minute walk - distance, fee	Endurance
Locomotion - 4-meter walk - Usual Pace - time, seconds	Locomotion
Dexterity - 9 Hole Pegboard - Dominant hand score	Dexterity
Dexterity - 9 Hole Pegboard - NonDominant hand score	Dexterity NDom
Upper Extremity Strength - Grip Strength - Dominant Hand, pounds	Strength
Upper Extremity Strength - Grip Strength - NonDominant Hand, pounds	Strength NDom

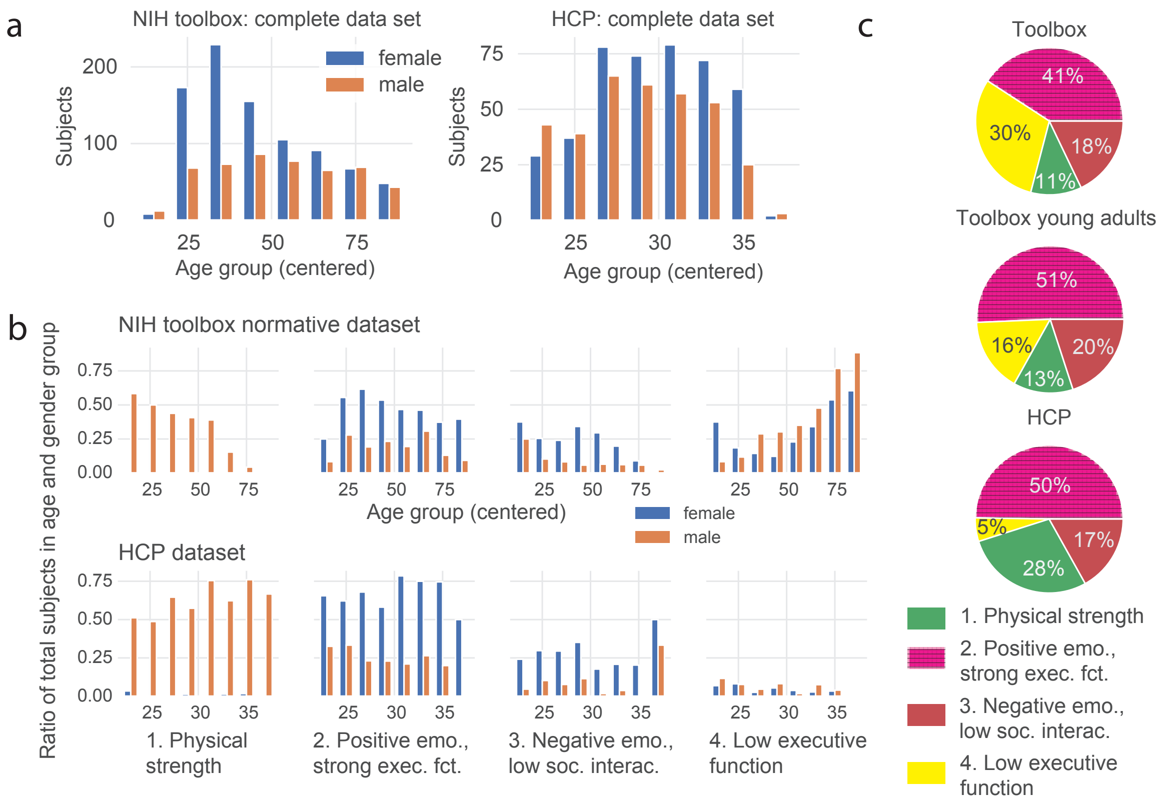

Figure S5 – Cluster composition is associated with age and gender and is stable across the two datasets.

The distribution of these variables is consistent along the clusters and reflects the underlying nature of their composition. **a:** Distribution of the NIH normative and HCP dataset according to age and gender. Here, NIH normative dataset subjects are binned into 10-year, HCP ones into 2-year age groups. **b:** Age and gender relative frequency relative for each of the four clusters in both datasets. A blue bar at 0.2 means that 20% of females in the corresponding age cluster are contained in this cluster. The y axis describes the fraction of subjects in the age and gender bracket belonging to this cluster. It is absolute, so bar heights can be compared across clusters. **c:** The clusters present similar proportionate sizes and structure when comparing the two datasets. A shift from cluster 4 (low executive and cognitive function) to cluster 1 (physical strength) is observed in the population as a results of gender distribution differences between the datasets.

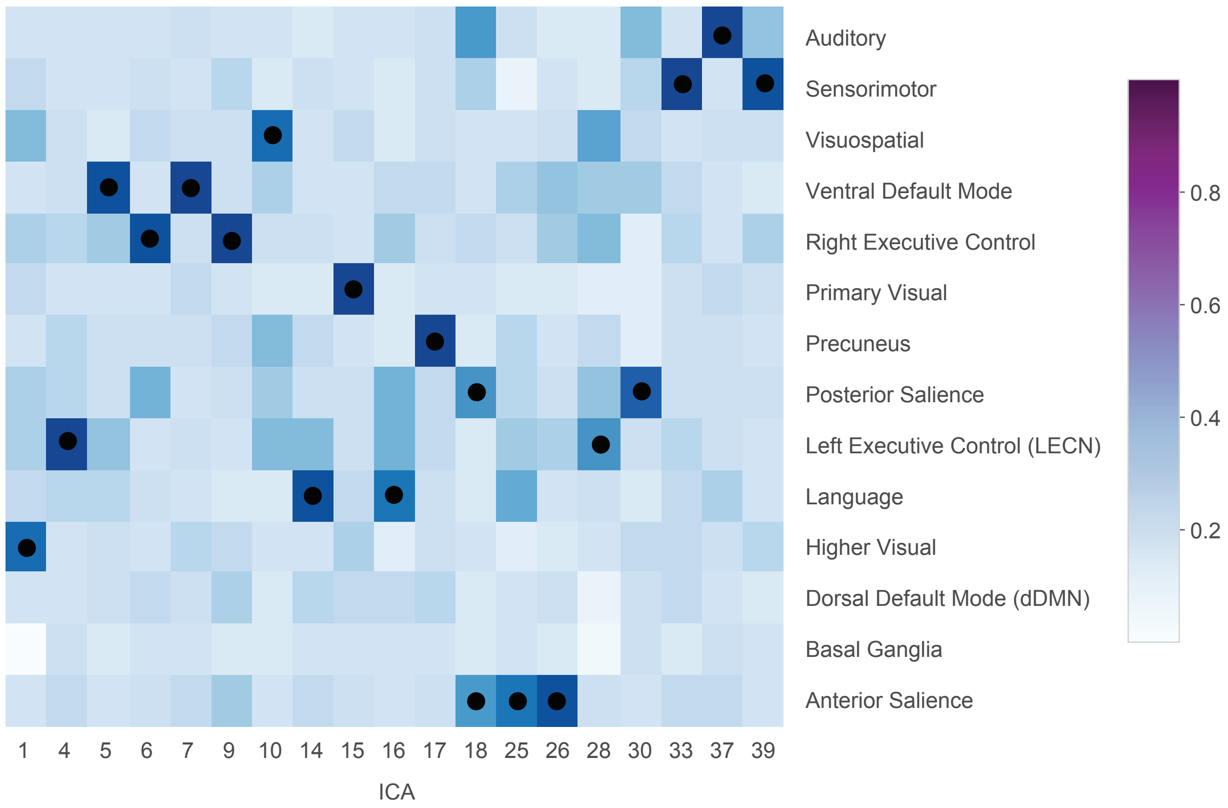

Figure S6 – Mapping the ICAs from the HCP data to brain ROIs from a public atlas (Shirer et al., 2012; Altmann et al., 2015) by correlating them. Darker colors correspond to stronger correlations. The black dot refers to the highest value per ICA, which we used to describe that ICA.

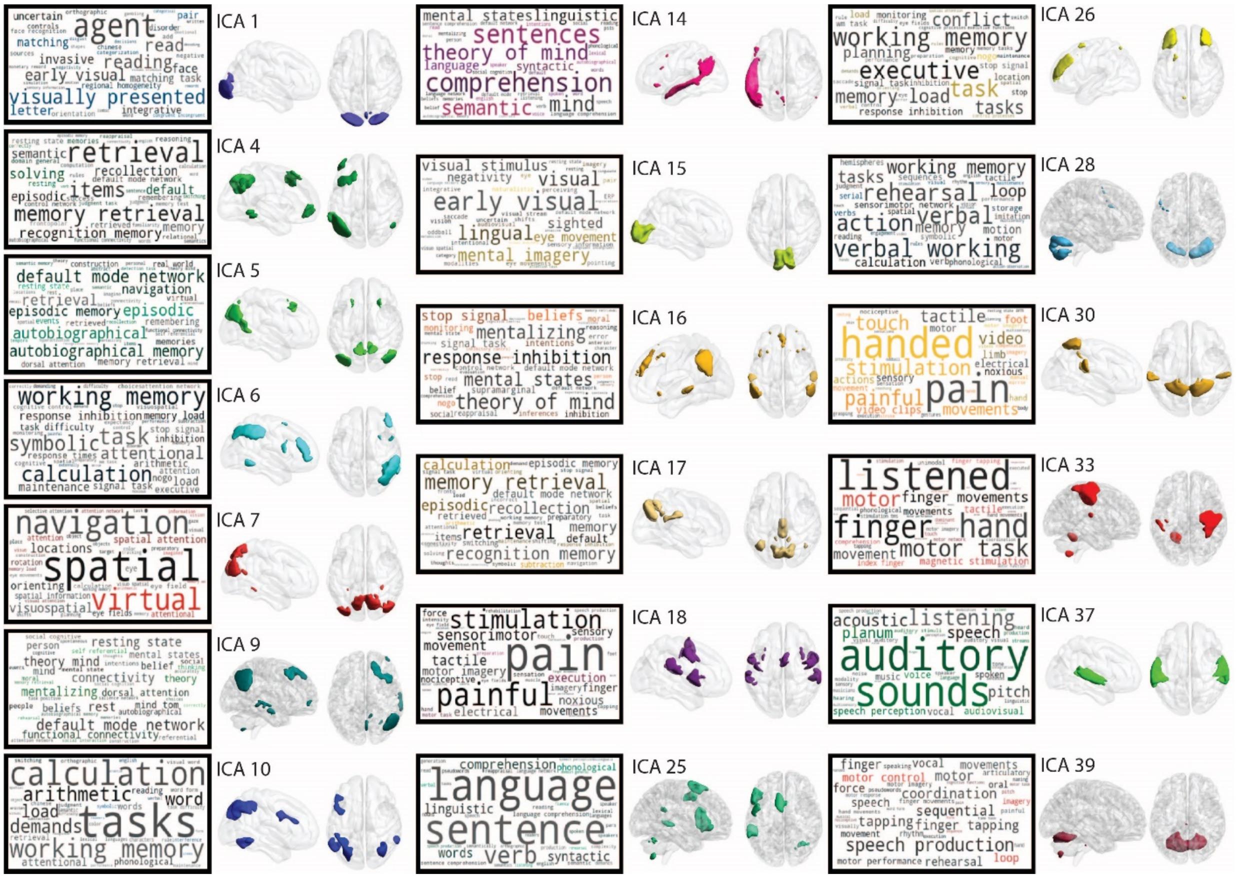

Figure S7 – Top semantic associations (word clouds) of brain large scale networks independent components.

Neurosynth results for brain regions involved in our analysis. Independent component analysis (ICA) from by the HCP consortium (dimensionality = 50) included in the final model were characterized using the Neurosynth. The size of a word in each word cloud is proportional to the strength of loading on the corresponding topic.

References

Altmann, A., Ng, B., Landau, S.M., Jagust, W.J., and Greicius, M.D. (2015). Regional brain hypometabolism is unrelated to regional amyloid plaque burden. *Brain* 138(12)**,** 3734-3746.

Eisenberg, I.W., Bissett, P.G., Enkavi, A.Z., Li, J., MacKinnon, D.P., Marsch, L.A., et al. (2019). Uncovering the structure of self-regulation through data-driven ontology discovery. *Nature communications* 10(1)**,** 1-13.

Shirer, W.R., Ryali, S., Rykhlevskaia, E., Menon, V., and Greicius, M.D. (2012). Decoding subject-driven cognitive states with whole-brain connectivity patterns. *Cerebral cortex* 22(1)**,** 158-165.
